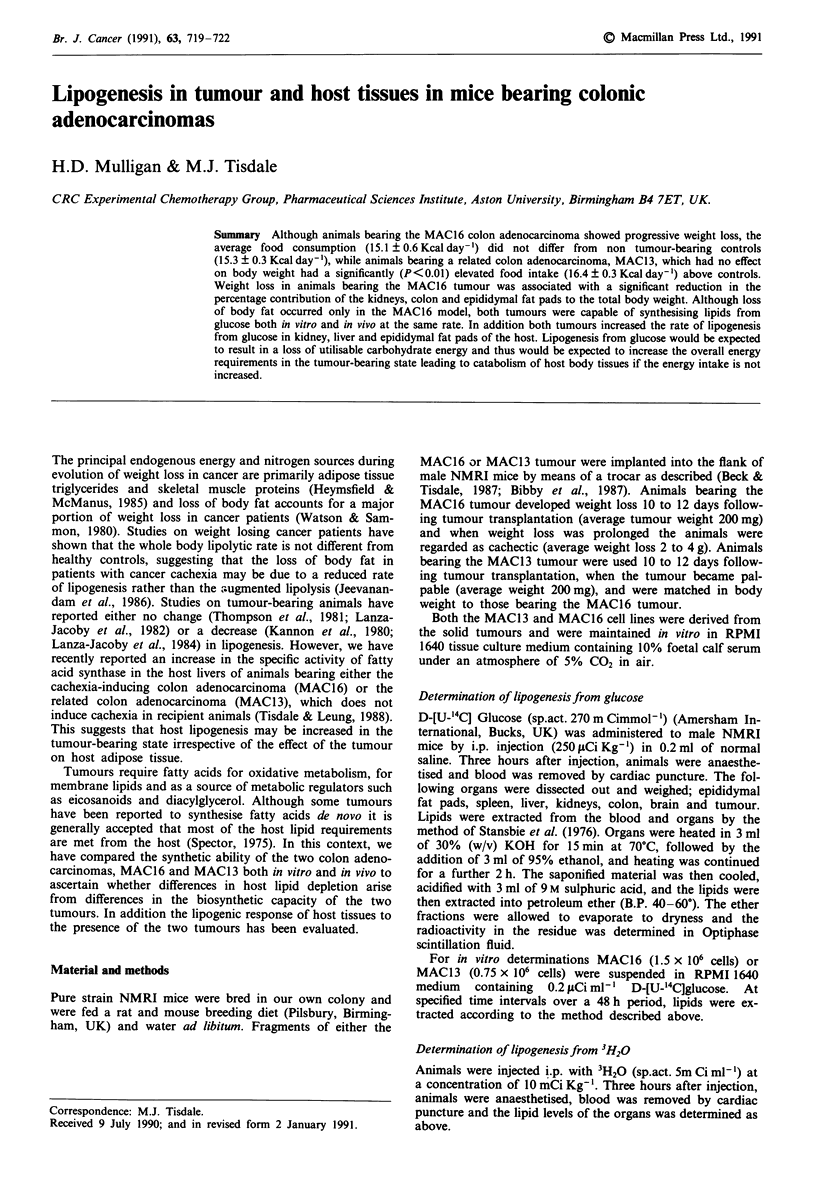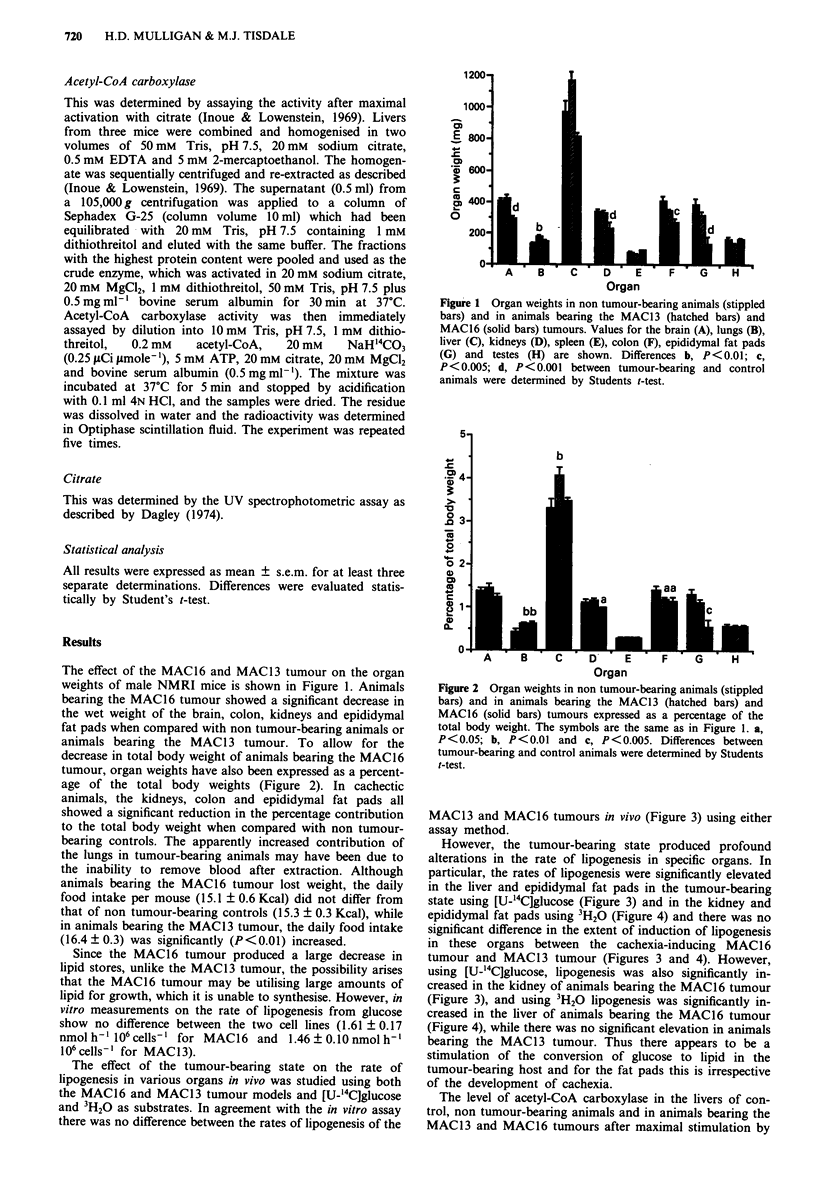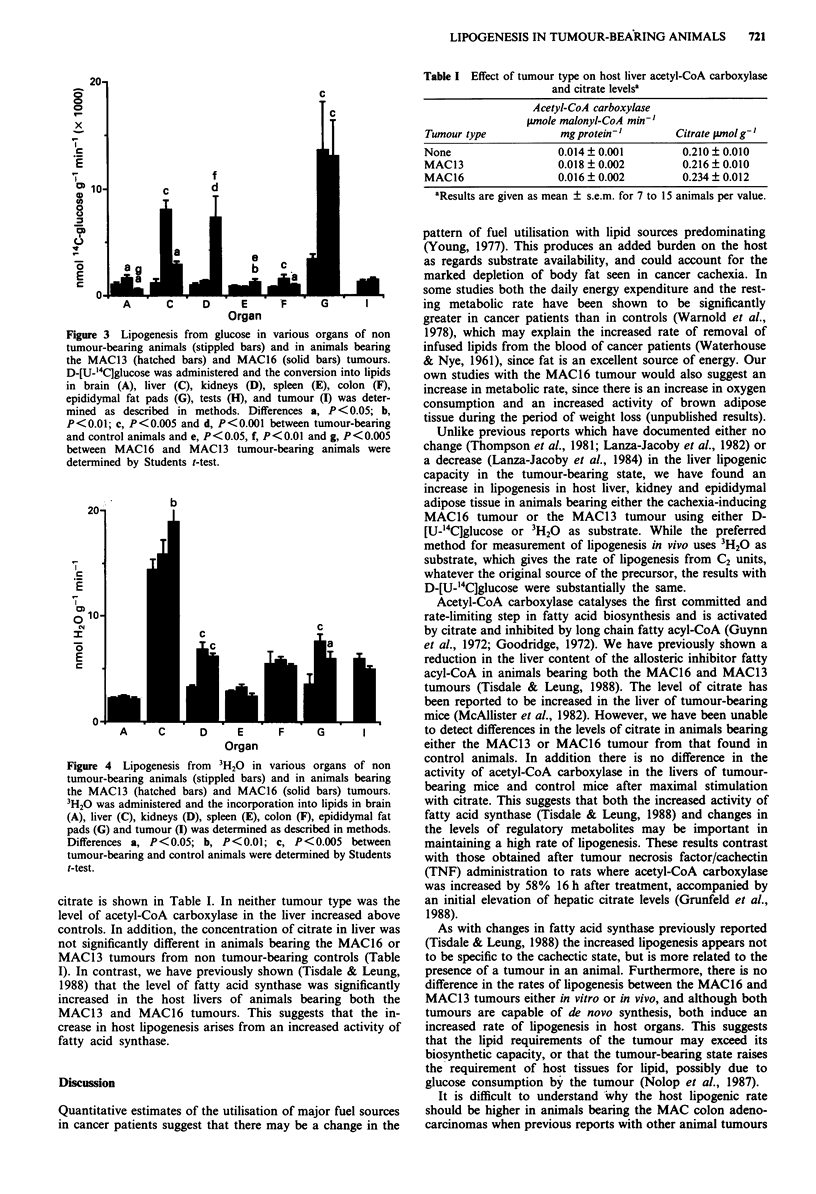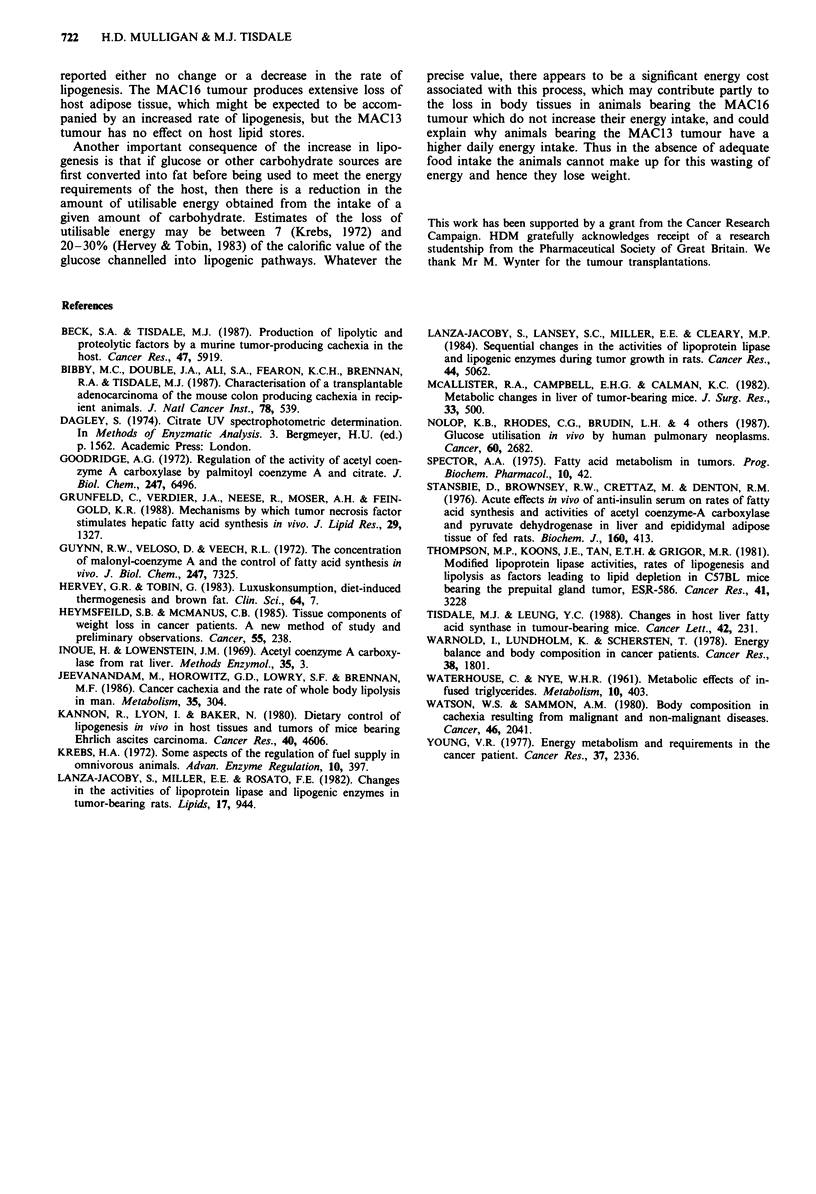# Lipogenesis in tumour and host tissues in mice bearing colonic adenocarcinomas.

**DOI:** 10.1038/bjc.1991.162

**Published:** 1991-05

**Authors:** H. D. Mulligan, M. J. Tisdale

**Affiliations:** Pharmaceutical Sciences Institute, Aston University, Birmingham, UK.

## Abstract

Although animals bearing the MAC16 colon adenocarcinoma showed progressive weight loss, the average food consumption (15.1 +/- 0.6 Kcal day-1) did not differ from non tumour-bearing controls (15.3 +/- 0.3 Kcal day-1), while animals bearing a related colon adenocarcinoma, MAC13, which had no effect on body weight had a significantly (P less than 0.01) elevated food intake (16.4 +/- 0.3 Kcal day-1) above controls. Weight loss in animals bearing the MAC16 tumour was associated with a significant reduction in the percentage contribution of the kidneys, colon and epididymal fat pads to the total body weight. Although loss of body fat occurred only in the MAC16 model, both tumours were capable of synthesising lipids from glucose both in vitro and in vivo at the same rate. In addition both tumours increased the rate of lipogenesis from glucose in kidney, liver and epididymal fat pads of the host. Lipogenesis from glucose would be expected to result in a loss of utilisable carbohydrate energy and thus would be expected to increase the overall energy requirements in the tumour-bearing state leading to catabolism of host body tissues if the energy intake is not increased.


Br. J. Cancer (1991), 63, 719-722 © Macmillan Press Ltd., 1991

### Lipogenesis in tumour and host tissues in mice bearing colonic adenocarcinomas

H.D. Mulligan & M.J. Tisdale

*CRC Experimental Chemotherapy Group, Pharmaceutical Sciences Institute, Aston University, Birmingham B4 7ET, UK.*

**Summary** Although animals bearing the MAC16 colon adenocarcinoma showed progressive weight loss, the average food consumption ($15.1 \pm 0.6$ Kcal day$^{-1}$) did not differ from non tumour-bearing controls ($15.3 \pm 0.3$ Kcal day$^{-1}$), while animals bearing a related colon adenocarcinoma, MAC13, which had no effect on body weight had a significantly ($P < 0.01$) elevated food intake ($16.4 \pm 0.3$ Kcal day$^{-1}$) above controls. Weight loss in animals bearing the MAC16 tumour was associated with a significant reduction in the percentage contribution of the kidneys, colon and epididymal fat pads to the total body weight. Although loss of body fat occurred only in the MAC16 model, both tumours were capable of synthesising lipids from glucose both *in vitro* and *in vivo* at the same rate. In addition both tumours increased the rate of lipogenesis from glucose in kidney, liver and epididymal fat pads of the host. Lipogenesis from glucose would be expected to result in a loss of utilisable carbohydrate energy and thus would be expected to increase the overall energy requirements in the tumour-bearing state leading to catabolism of host body tissues if the energy intake is not increased.

The principal endogenous energy and nitrogen sources during evolution of weight loss in cancer are primarily adipose tissue triglycerides and skeletal muscle proteins (Heymsfield & McManus, 1985) and loss of body fat accounts for a major portion of weight loss in cancer patients (Watson & Sammon, 1980). Studies on weight losing cancer patients have shown that the whole body lipolytic rate is not different from healthy controls, suggesting that the loss of body fat in patients with cancer cachexia may be due to a reduced rate of lipogenesis rather than the augmented lipolysis (Jeevanandam *et al.*, 1986). Studies on tumour-bearing animals have reported either no change (Thompson *et al.*, 1981; Lanza-Jacoby *et al.*, 1982) or a decrease (Kannon *et al.*, 1980; Lanza-Jacoby *et al.*, 1984) in lipogenesis. However, we have recently reported an increase in the specific activity of fatty acid synthase in the host livers of animals bearing either the cachexia-inducing colon adenocarcinoma (MAC16) or the related colon adenocarcinoma (MAC13), which does not induce cachexia in recipient animals (Tisdale & Leung, 1988). This suggests that host lipogenesis may be increased in the tumour-bearing state irrespective of the effect of the tumour on host adipose tissue.

Tumours require fatty acids for oxidative metabolism, for membrane lipids and as a source of metabolic regulators such as eicosanoids and diacylglycerol. Although some tumours have been reported to synthesise fatty acids *de novo* it is generally accepted that most of the host lipid requirements are met from the host (Spector, 1975). In this context, we have compared the synthetic ability of the two colon adenocarcinomas, MAC16 and MAC13 both *in vitro* and *in vivo* to ascertain whether differences in host lipid depletion arise from differences in the biosynthetic capacity of the two tumours. In addition the lipogenic response of host tissues to the presence of the two tumours has been evaluated.

### Material and methods

Pure strain NMRI mice were bred in our own colony and were fed a rat and mouse breeding diet (Pilsbury, Birmingham, UK) and water *ad libitum*. Fragments of either the MAC16 or MAC13 tumour were implanted into the flank of male NMRI mice by means of a trocar as described (Beck & Tisdale, 1987; Bibby *et al.*, 1987). Animals bearing the MAC16 tumour developed weight loss 10 to 12 days following tumour transplantation (average tumour weight 200 mg) and when weight loss was prolonged the animals were regarded as cachectic (average weight loss 2 to 4 g). Animals bearing the MAC13 tumour were used 10 to 12 days following tumour transplantation, when the tumour became palpable (average weight 200 mg), and were matched in body weight to those bearing the MAC16 tumour.

Both the MAC13 and MAC16 cell lines were derived from the solid tumours and were maintained *in vitro* in RPMI 1640 tissue culture medium containing 10% foetal calf serum under an atmosphere of 5% $CO_2$ in air.

### Determination of lipogenesis from glucose

D-[U-$^{14}$C] Glucose (sp.act. 270 m Cimmol$^{-1}$) (Amersham International, Bucks, UK) was administered to male NMRI mice by i.p. injection (250 $\mu$Ci Kg$^{-1}$) in 0.2 ml of normal saline. Three hours after injection, animals were anaesthetised and blood was removed by cardiac puncture. The following organs were dissected out and weighed; epididymal fat pads, spleen, liver, kidneys, colon, brain and tumour. Lipids were extracted from the blood and organs by the method of Stansbie *et al.* (1976). Organs were heated in 3 ml of 30% (w/v) KOH for 15 min at 70°C, followed by the addition of 3 ml of 95% ethanol, and heating was continued for a further 2 h. The saponified material was then cooled, acidified with 3 ml of 9 M sulphuric acid, and the lipids were then extracted into petroleum ether (B.P. 40-60°). The ether fractions were allowed to evaporate to dryness and the radioactivity in the residue was determined in Optiphase scintillation fluid.

For *in vitro* determinations MAC16 ($1.5 \times 10^6$ cells) or MAC13 ($0.75 \times 10^6$ cells) were suspended in RPMI 1640 medium containing 0.2 $\mu$Ci ml$^{-1}$ D-[U-$^{14}$C]glucose. At specified time intervals over a 48 h period, lipids were extracted according to the method described above.

### Determination of lipogenesis from $^3H_2O$

Animals were injected i.p. with $^3H_2O$ (sp.act. 5m Ci ml$^{-1}$) at a concentration of 10 mCi Kg$^{-1}$. Three hours after injection, animals were anaesthetised, blood was removed by cardiac puncture and the lipid levels of the organs was determined as above.

Correspondence: M.J. Tisdale.
Received 9 July 1990; and in revised form 2 January 1991.

*Acetyl-CoA carboxylase*

This was determined by assaying the activity after maximal activation with citrate (Inoue & Lowenstein, 1969). Livers from three mice were combined and homogenised in two volumes of 50 mM Tris, pH 7.5, 20 mM sodium citrate, 0.5 mM EDTA and 5 mM 2-mercaptoethanol. The homogenate was sequentially centrifuged and re-extracted as described (Inoue & Lowenstein, 1969). The supernatant (0.5 ml) from a 105,000 *g* centrifugation was applied to a column of Sephadex G-25 (column volume 10 ml) which had been equilibrated with 20 mM Tris, pH 7.5 containing 1 mM dithiothreitol and eluted with the same buffer. The fractions with the highest protein content were pooled and used as the crude enzyme, which was activated in 20 mM sodium citrate, 20 mM $MgCl_2$, 1 mM dithiothreitol, 50 mM Tris, pH 7.5 plus 0.5 mg $ml^{-1}$ bovine serum albumin for 30 min at 37°C. Acetyl-CoA carboxylase activity was then immediately assayed by dilution into 10 mM Tris, pH 7.5, 1 mM dithiothreitol, 0.2 mM acetyl-CoA, 20 mM $NaH^{14}CO_3$ (0.25 μCi $\mu mole^{-1}$), 5 mM ATP, 20 mM citrate, 20 mM $MgCl_2$ and bovine serum albumin (0.5 mg $ml^{-1}$). The mixture was incubated at 37°C for 5 min and stopped by acidification with 0.1 ml 4N HCl, and the samples were dried. The residue was dissolved in water and the radioactivity was determined in Optiphase scintillation fluid. The experiment was repeated five times.

*Citrate*

This was determined by the UV spectrophotometric assay as described by Dagley (1974).

*Statistical analysis*

All results were expressed as mean ± s.e.m. for at least three separate determinations. Differences were evaluated statistically by Student's *t*-test.

### Results

The effect of the MAC16 and MAC13 tumour on the organ weights of male NMRI mice is shown in Figure 1. Animals bearing the MAC16 tumour showed a significant decrease in the wet weight of the brain, colon, kidneys and epididymal fat pads when compared with non tumour-bearing animals or animals bearing the MAC13 tumour. To allow for the decrease in total body weight of animals bearing the MAC16 tumour, organ weights have also been expressed as a percentage of the total body weights (Figure 2). In cachectic animals, the kidneys, colon and epididymal fat pads all showed a significant reduction in the percentage contribution to the total body weight when compared with non tumour-bearing controls. The apparently increased contribution of the lungs in tumour-bearing animals may have been due to the inability to remove blood after extraction. Although animals bearing the MAC16 tumour lost weight, the daily food intake per mouse (15.1 ± 0.6 Kcal) did not differ from that of non tumour-bearing controls (15.3 ± 0.3 Kcal), while in animals bearing the MAC13 tumour, the daily food intake (16.4 ± 0.3) was significantly ($P < 0.01$) increased.

Since the MAC16 tumour produced a large decrease in lipid stores, unlike the MAC13 tumour, the possibility arises that the MAC16 tumour may be utilising large amounts of lipid for growth, which it is unable to synthesise. However, *in vitro* measurements on the rate of lipogenesis from glucose show no difference between the two cell lines (1.61 ± 0.17 nmol $h^{-1}$ $10^6$ $cells^{-1}$ for MAC16 and 1.46 ± 0.10 nmol $h^{-1}$ $10^6$ $cells^{-1}$ for MAC13).

The effect of the tumour-bearing state on the rate of lipogenesis in various organs *in vivo* was studied using both the MAC16 and MAC13 tumour models and [U-$^{14}$C]glucose and $^3H_2O$ as substrates. In agreement with the *in vitro* assay there was no difference between the rates of lipogenesis of the MAC13 and MAC16 tumours *in vivo* (Figure 3) using either assay method.

**Figure 1** Organ weights in non tumour-bearing animals (stippled bars) and in animals bearing the MAC13 (hatched bars) and MAC16 (solid bars) tumours. Values for the brain (**A**), lungs (**B**), liver (**C**), kidneys (**D**), spleen (**E**), colon (**F**), epididymal fat pads (**G**) and testes (**H**) are shown. Differences **b**, $P < 0.01$; **c**, $P < 0.005$; **d**, $P < 0.001$ between tumour-bearing and control animals were determined by Students *t*-test.

**Figure 2** Organ weights in non tumour-bearing animals (stippled bars) and in animals bearing the MAC13 (hatched bars) and MAC16 (solid bars) tumours expressed as a percentage of the total body weight. The symbols are the same as in Figure 1. **a**, $P < 0.05$; **b**, $P < 0.01$ and **c**, $P < 0.005$. Differences between tumour-bearing and control animals were determined by Students *t*-test.

However, the tumour-bearing state produced profound alterations in the rate of lipogenesis in specific organs. In particular, the rates of lipogenesis were significantly elevated in the liver and epididymal fat pads in the tumour-bearing state using [U-$^{14}$C]glucose (Figure 3) and in the kidney and epididymal fat pads using $^3H_2O$ (Figure 4) and there was no significant difference in the extent of induction of lipogenesis in these organs between the cachexia-inducing MAC16 tumour and MAC13 tumour (Figures 3 and 4). However, using [U-$^{14}$C]glucose, lipogenesis was also significantly increased in the kidney of animals bearing the MAC16 tumour (Figure 3), and using $^3H_2O$ lipogenesis was significantly increased in the liver of animals bearing the MAC16 tumour (Figure 4), while there was no significant elevation in animals bearing the MAC13 tumour. Thus there appears to be a stimulation of the conversion of glucose to lipid in the tumour-bearing host and for the fat pads this is irrespective of the development of cachexia.

The level of acetyl-CoA carboxylase in the livers of control, non tumour-bearing animals and in animals bearing the MAC13 and MAC16 tumours after maximal stimulation by

**Figure 3** Lipogenesis from glucose in various organs of non tumour-bearing animals (stippled bars) and in animals bearing the MAC13 (hatched bars) and MAC16 (solid bars) tumours. D-[U-$^{14}$C]glucose was administered and the conversion into lipids in brain (A), liver (C), kidneys (D), spleen (E), colon (F), epididymal fat pads (G), tests (H), and tumour (I) was determined as described in methods. Differences **a**, $P<0.05$; **b**, $P<0.01$; **c**, $P<0.005$ and **d**, $P<0.001$ between tumour-bearing and control animals and **e**, $P<0.05$, **f**, $P<0.01$ and **g**, $P<0.005$ between MAC16 and MAC13 tumour-bearing animals were determined by Students *t*-test.

**Figure 4** Lipogenesis from $^3H_2O$ in various organs of non tumour-bearing animals (stippled bars) and in animals bearing the MAC13 (hatched bars) and MAC16 (solid bars) tumours. $^3H_2O$ was administered and the incorporation into lipids in brain (A), liver (C), kidneys (D), spleen (E), colon (F), epididymal fat pads (G) and tumour (I) was determined as described in methods. Differences **a**, $P<0.05$; **b**, $P<0.01$; **c**, $P<0.005$ between tumour-bearing and control animals were determined by Students *t*-test.

citrate is shown in Table I. In neither tumour type was the level of acetyl-CoA carboxylase in the liver increased above controls. In addition, the concentration of citrate in liver was not significantly different in animals bearing the MAC16 or MAC13 tumours from non tumour-bearing controls (Table I). In contrast, we have previously shown (Tisdale & Leung, 1988) that the level of fatty acid synthase was significantly increased in the host livers of animals bearing both the MAC13 and MAC16 tumours. This suggests that the increase in host lipogenesis arises from an increased activity of fatty acid synthase.

### Discussion

Quantitative estimates of the utilisation of major fuel sources in cancer patients suggest that there may be a change in the pattern of fuel utilisation with lipid sources predominating (Young, 1977). This produces an added burden on the host as regards substrate availability, and could account for the marked depletion of body fat seen in cancer cachexia. In some studies both the daily energy expenditure and the resting metabolic rate have been shown to be significantly greater in cancer patients than in controls (Warnold *et al.*, 1978), which may explain the increased rate of removal of infused lipids from the blood of cancer patients (Waterhouse & Nye, 1961), since fat is an excellent source of energy. Our own studies with the MAC16 tumour would also suggest an increase in metabolic rate, since there is an increase in oxygen consumption and an increased activity of brown adipose tissue during the period of weight loss (unpublished results).

Unlike previous reports which have documented either no change (Thompson *et al.*, 1981; Lanza-Jacoby *et al.*, 1982) or a decrease (Lanza-Jacoby *et al.*, 1984) in the liver lipogenic capacity in the tumour-bearing state, we have found an increase in lipogenesis in host liver, kidney and epididymal adipose tissue in animals bearing either the cachexia-inducing MAC16 tumour or the MAC13 tumour using either D-[U-$^{14}$C]glucose or $^3H_2O$ as substrate. While the preferred method for measurement of lipogenesis *in vivo* uses $^3H_2O$ as substrate, which gives the rate of lipogenesis from $C_2$ units, whatever the original source of the precursor, the results with D-[U-$^{14}$C]glucose were substantially the same.

Acetyl-CoA carboxylase catalyses the first committed and rate-limiting step in fatty acid biosynthesis and is activated by citrate and inhibited by long chain fatty acyl-CoA (Guynn *et al.*, 1972; Goodridge, 1972). We have previously shown a reduction in the liver content of the allosteric inhibitor fatty acyl-CoA in animals bearing both the MAC16 and MAC13 tumours (Tisdale & Leung, 1988). The level of citrate has been reported to be increased in the liver of tumour-bearing mice (McAllister *et al.*, 1982). However, we have been unable to detect differences in the levels of citrate in animals bearing either the MAC13 or MAC16 tumour from that found in control animals. In addition there is no difference in the activity of acetyl-CoA carboxylase in the livers of tumour-bearing mice and control mice after maximal stimulation with citrate. This suggests that both the increased activity of fatty acid synthase (Tisdale & Leung, 1988) and changes in the levels of regulatory metabolites may be important in maintaining a high rate of lipogenesis. These results contrast with those obtained after tumour necrosis factor/cachectin (TNF) administration to rats where acetyl-CoA carboxylase was increased by 58% 16 h after treatment, accompanied by an initial elevation of hepatic citrate levels (Grunfeld *et al.*, 1988).

As with changes in fatty acid synthase previously reported (Tisdale & Leung, 1988) the increased lipogenesis appears not to be specific to the cachectic state, but is more related to the presence of a tumour in an animal. Furthermore, there is no difference in the rates of lipogenesis between the MAC16 and MAC13 tumours either *in vitro* or *in vivo*, and although both tumours are capable of *de novo* synthesis, both induce an increased rate of lipogenesis in host organs. This suggests that the lipid requirements of the tumour may exceed its biosynthetic capacity, or that the tumour-bearing state raises the requirement of host tissues for lipid, possibly due to glucose consumption by the tumour (Nolop *et al.*, 1987).

It is difficult to understand why the host lipogenic rate should be higher in animals bearing the MAC colon adenocarcinomas when previous reports with other animal tumours

**Table I** Effect of tumour type on host liver acetyl-CoA carboxylase and citrate levels[a]

| *Tumour type* | *Acetyl-CoA carboxylase µmole malonyl-CoA min$^{-1}$ mg protein$^{-1}$* | *Citrate µmol g$^{-1}$* |
|---|---|---|
| None | 0.014 ± 0.001 | 0.210 ± 0.010 |
| MAC13 | 0.018 ± 0.002 | 0.216 ± 0.010 |
| MAC16 | 0.016 ± 0.002 | 0.234 ± 0.012 |

[a]Results are given as mean ± s.e.m. for 7 to 15 animals per value.

reported either no change or a decrease in the rate of lipogenesis. The MAC16 tumour produces extensive loss of host adipose tissue, which might be expected to be accompanied by an increased rate of lipogenesis, but the MAC13 tumour has no effect on host lipid stores.

Another important consequence of the increase in lipogenesis is that if glucose or other carbohydrate sources are first converted into fat before being used to meet the energy requirements of the host, then there is a reduction in the amount of utilisable energy obtained from the intake of a given amount of carbohydrate. Estimates of the loss of utilisable energy may be between 7 (Krebs, 1972) and 20–30% (Hervey & Tobin, 1983) of the calorific value of the glucose channelled into lipogenic pathways. Whatever the precise value, there appears to be a significant energy cost associated with this process, which may contribute partly to the loss in body tissues in animals bearing the MAC16 tumour which do not increase their energy intake, and could explain why animals bearing the MAC13 tumour have a higher daily energy intake. Thus in the absence of adequate food intake the animals cannot make up for this wasting of energy and hence they lose weight.

This work has been supported by a grant from the Cancer Research Campaign. HDM gratefully acknowledges receipt of a research studentship from the Pharmaceutical Society of Great Britain. We thank Mr M. Wynter for the tumour transplantations.

### References

BECK, S.A. & TISDALE, M.J. (1987). Production of lipolytic and proteolytic factors by a murine tumor-producing cachexia in the host. *Cancer Res.*, **47**, 5919.

BIBBY, M.C., DOUBLE, J.A., ALI, S.A., FEARON, K.C.H., BRENNAN, R.A. & TISDALE, M.J. (1987). Characterisation of a transplantable adenocarcinoma of the mouse colon producing cachexia in recipient animals. *J. Natl Cancer Inst.*, **78**, 539.

DAGLEY, S. (1974). Citrate UV spectrophotometric determination. In *Methods of Enyzmatic Analysis*. 3. Bergmeyer, H.U. (ed.) p. 1562. Academic Press: London.

GOODRIDGE, A.G. (1972). Regulation of the activity of acetyl coenzyme A carboxylase by palmitoyl coenzyme A and citrate. *J. Biol. Chem.*, **247**, 6496.

GRUNFELD, C., VERDIER, J.A., NEESE, R., MOSER, A.H. & FEINGOLD, K.R. (1988). Mechanisms by which tumor necrosis factor stimulates hepatic fatty acid synthesis *in vivo*. *J. Lipid Res.*, **29**, 1327.

GUYNN, R.W., VELOSO, D. & VEECH, R.L. (1972). The concentration of malonyl-coenzyme A and the control of fatty acid synthesis *in vivo*. *J. Biol. Chem.*, **247**, 7325.

HERVEY, G.R. & TOBIN, G. (1983). Luxuskonsumption, diet-induced thermogenesis and brown fat. *Clin. Sci.*, **64**, 7.

HEYMSFEILD, S.B. & MCMANUS, C.B. (1985). Tissue components of weight loss in cancer patients. A new method of study and preliminary observations. *Cancer*, **55**, 238.

INOUE, H. & LOWENSTEIN, J.M. (1969). Acetyl coenzyme A carboxylase from rat liver. *Methods Enzymol.*, **35**, 3.

JEEVANANDAM, M., HOROWITZ, G.D., LOWRY, S.F. & BRENNAN, M.F. (1986). Cancer cachexia and the rate of whole body lipolysis in man. *Metabolism*, **35**, 304.

KANNON, R., LYON, I. & BAKER, N. (1980). Dietary control of lipogenesis *in vivo* in host tissues and tumors of mice bearing Ehrlich ascites carcinoma. *Cancer Res.*, **40**, 4606.

KREBS, H.A. (1972). Some aspects of the regulation of fuel supply in omnivorous animals. *Advan. Enzyme Regulation*, **10**, 397.

LANZA-JACOBY, S., MILLER, E.E. & ROSATO, F.E. (1982). Changes in the activities of lipoprotein lipase and lipogenic enzymes in tumor-bearing rats. *Lipids*, **17**, 944.

LANZA-JACOBY, S., LANSEY, S.C., MILLER, E.E. & CLEARY, M.P. (1984). Sequential changes in the activities of lipoprotein lipase and lipogenic enzymes during tumor growth in rats. *Cancer Res.*, **44**, 5062.

MCALLISTER, R.A., CAMPBELL, E.H.G. & CALMAN, K.C. (1982). Metabolic changes in liver of tumor-bearing mice. *J. Surg. Res.*, **33**, 500.

NOLOP, K.B., RHODES, C.G., BRUDIN, L.H. & 4 others (1987). Glucose utilisation *in vivo* by human pulmonary neoplasms. *Cancer*, **60**, 2682.

SPECTOR, A.A. (1975). Fatty acid metabolism in tumors. *Prog. Biochem. Pharmacol.*, **10**, 42.

STANSBIE, D., BROWNSEY, R.W., CRETTAZ, M. & DENTON, R.M. (1976). Acute effects *in vivo* of anti-insulin serum on rates of fatty acid synthesis and activities of acetyl coenzyme-A carboxylase and pyruvate dehydrogenase in liver and epididymal adipose tissue of fed rats. *Biochem. J.*, **160**, 413.

THOMPSON, M.P., KOONS, J.E., TAN, E.T.H. & GRIGOR, M.R. (1981). Modified lipoprotein lipase activities, rates of lipogenesis and lipolysis as factors leading to lipid depletion in C57BL mice bearing the prepuital gland tumor, ESR-586. *Cancer Res.*, **41**, 3228

TISDALE, M.J. & LEUNG, Y.C. (1988). Changes in host liver fatty acid synthase in tumour-bearing mice. *Cancer Lett.*, **42**, 231.

WARNOLD, I., LUNDHOLM, K. & SCHERSTEN, T. (1978). Energy balance and body composition in cancer patients. *Cancer Res.*, **38**, 1801.

WATERHOUSE, C. & NYE, W.H.R. (1961). Metabolic effects of infused triglycerides. *Metabolism*, **10**, 403.

WATSON, W.S. & SAMMON, A.M. (1980). Body composition in cachexia resulting from malignant and non-malignant diseases. *Cancer*, **46**, 2041.

YOUNG, V.R. (1977). Energy metabolism and requirements in the cancer patient. *Cancer Res.*, **37**, 2336.